# Supplementary material for: HIVprotI: an integrated web based platform for prediction and design of HIV proteins inhibitors
Source: J Cheminform. 2018 Mar 9;10:12. doi: 10.1186/s13321-018-0266-y (PMC5845081; doi:10.1186/s13321-018-0266-y)
Supplement: Supplementary file 2 — Additional file 2. Source code of HIVProtI web server. [file 13321_2018_266_MOESM2_ESM.zip › HIVprotI_Source-code/hivprotI/weka-3-6-6/doc/weka/classifiers/lazy/kstar/KStarCache.html]

KStarCache


---


|  |  |  |
| --- | --- | --- |
| **Overview** \| **Package** \| **Class** \| **Tree** \| **Deprecated** \| **Index** \| **Help** \| **Weka's home** | |  |
| PREV CLASS   **NEXT CLASS** | **FRAMES**    **NO FRAMES**     **All Classes** |  |
| SUMMARY: NESTED \| FIELD \| CONSTR \| METHOD | DETAIL: FIELD \| CONSTR \| METHOD |  |


---


## weka.classifiers.lazy.kstar Class KStarCache

```
java.lang.Object
  weka.classifiers.lazy.kstar.KStarCache
```

**All Implemented Interfaces:**: java.io.Serializable, RevisionHandler

---

``` public class KStarCache extends java.lang.Object implements java.io.Serializable, RevisionHandler ```

A class representing the caching system used to keep track of each attribute
value and its corresponding scale factor or stop parameter.

**Version:**
:   $Revision: 1.11 $

**Author:**
:   Len Trigg (len@reeltwo.com), Abdelaziz Mahoui (am14@cs.waikato.ac.nz)

**See Also:**: Serialized Form

---

| **Nested Class Summary** | |
| --- | --- |
| `class` | `KStarCache.CacheTable`             A custom hashtable class to support the caching system. |
| `class` | `KStarCache.TableEntry`             Hashtable collision list. |


**Constructor Summary**

`KStarCache()`


| **Method Summary** | |
| --- | --- |
| `boolean` | `containsKey(double key)`             Checks if the specified key maps with an entry in the cache table |
| `KStarCache.TableEntry` | `getCacheValues(double key)`             Returns the values in the cache mapped by the specified key |
| `java.lang.String` | `getRevision()`             Returns the revision string. |
| `void` | `store(double key, double value, double pmiss)`             Stores the specified values in the cahce table for easy retrieval. |

**Methods inherited from class java.lang.Object**

`equals, getClass, hashCode, notify, notifyAll, toString, wait, wait, wait`

**Constructor Detail**

### KStarCache

```
public KStarCache()
```


**Method Detail**

### store

```
public void store(double key,
                  double value,
                  double pmiss)
```

:   Stores the specified values in the cahce table for easy retrieval.

    :   **Parameters:**: `key` - attribute value used key to lookup the cache table.: `value` - cache parameter: attribute scale/stop parameter.: `pmiss` - cache parameter: transformation probability to attribute with missing value.

---


### containsKey

```
public boolean containsKey(double key)
```

:   Checks if the specified key maps with an entry in the cache table

    :   **Parameters:**: `key` - the key to map with an entry in the hashtable.

---


### getCacheValues

```
public KStarCache.TableEntry getCacheValues(double key)
```

:   Returns the values in the cache mapped by the specified key

    :   **Parameters:**: `key` - the key used to retrieve the table entry.

---


### getRevision

```
public java.lang.String getRevision()
```

:   Returns the revision string.

    :   **Specified by:**: `getRevision` in interface `RevisionHandler`
    :   **Returns:**: the revision


---


|  |  |  |
| --- | --- | --- |
| **Overview** \| **Package** \| **Class** \| **Tree** \| **Deprecated** \| **Index** \| **Help** \| **Weka's home** | |  |
| PREV CLASS   **NEXT CLASS** | **FRAMES**    **NO FRAMES**     **All Classes** |  |
| SUMMARY: NESTED \| FIELD \| CONSTR \| METHOD | DETAIL: FIELD \| CONSTR \| METHOD |  |


---
